# Data on atherosclerosis specific antibody conjugation to nanoemulsions

**DOI:** 10.1016/j.dib.2017.10.058

**Published:** 2017-10-26

**Authors:** Geoffrey Prévot, Martine Duonor-Cérutti, Mélusine Larivière, Jeanny Laroche-Traineau, Marie Josée Jacobin-Valat, Philippe Barthélémy, Gisèle Clofent-Sanchez, Sylvie Crauste-Manciet

**Affiliations:** a Univ. Bordeaux, INSERM, U1212, CNRS UMR 5320, ARNA, ARN: Régulations Naturelle et Artificielle, ChemBioPharm, F-33000 Bordeaux, France; b CNRS UPS3044, Baculovirus et Thérapie, F-30380 Saint-Christol-lès-Alès, France; c Univ. Bordeaux, CNRS UMR 5536, CRMSB, Centre de Résonance Magnétique des Systèmes Biologiques, F-33000 Bordeaux, France

## Abstract

This article present data related to the publication entitled “Iron oxide core oil-in-water nanoemulsion as tracer for atherosclerosis MPI and MRI imaging” (Prévot et al., 2017) [1]. Herein we describe the engineering in the baculovirus-insect cell system and purification processes of the human scFv-Fc TEG4-2C antibody, specific of platelets within the atheroma plaque. For molecular targeting purpose, atheroma specific antibody was conjugated to nanoemulsions (NEs) using a heterobifunctional linker (DSPE-PEG-maleimide). Atheroma labelling was assayed by immunochemistry on arterial sections from rabbits.

**Specifications Table**

**Table:** 

Subject area	*Biology*
More specific subject area	*Antibody engineering*
Type of data	*Protocols and figures*
How data was acquired	*NE vesicle size was obtained by dynamic light scattering (DLS), maleimide quantification was performed by spectrophotometer*
Data format	*Analysed*
Experimental factors	*ScFv-Fc TEG4-2C was produced using the baculovirus-insect cell system then purified on protein A Sepharose*
Experimental features	Experimental description of TEG4 antibody engineering
Data source location	*Bordeaux, France*
Data accessibility	*The data are provided with this article*

**Value of the data**

• Engineering and purification processes of *scFv-Fc TEG4-2C* antibody for molecular targeting.
• Maleimide expression at NE vesicle confirmed by spectrophotometer prior antibody grafting.
• Evaluation of *in vitro* molecular targeting by immunohistochemistry.
• Colloidal stability of NE when stored at 4 °C.
• These data are valuable for the scientific community that work with antibodies.

## 1 Data

**Fig. 1:**
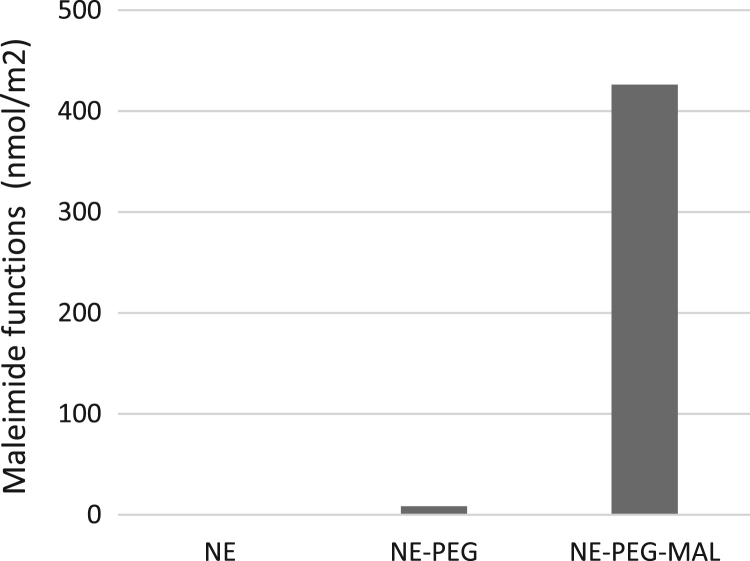
Maleimide quantification expressed at droplet surface of ungrafted emulsion (NE), emulsion formulated with PEG only (NE-PEG) and emulsion formulated with PEG-maleimide (NE-PEG-MAL).

**Fig. 2:**
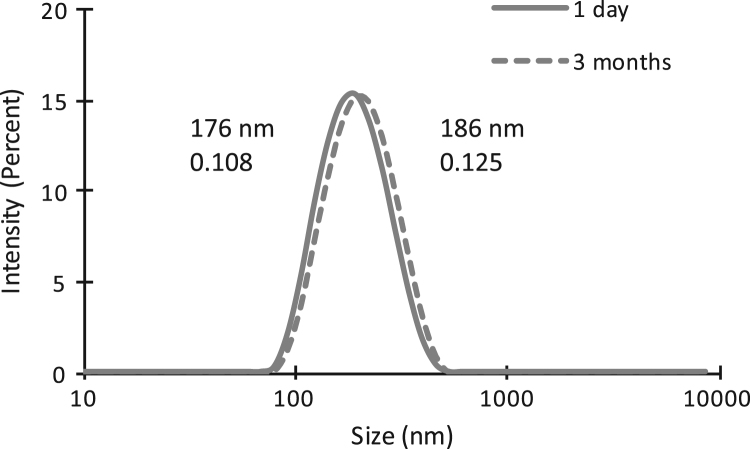
NE size distribution, on day one (continuous line) and 3 months after formulation (dashed line).

The dataset of this article provides information about the engineering and purification of a specific human antibody for molecular imaging of atheroma ​[1]. This human antibody was designed with two cysteines at the Fc site for site-specific grafting. Antibody bearing thiol functions was grafted to NEs surface using PEG-maleimide macromolecules. Fig. 1 displays the quantification of maleimide functions by spectrometric assays. Formulation colloidal stability was assayed during 3 months by dynamic light scattering (DLS) (Fig. 2).

## 2 Experimental design, materials and methods

### 2.1 Generation of recombinant baculovirus expressing scFv-Fc TEG4-2C

ScFv-Fc TEG4-2C was produced using the baculovirus-insect cell system. Briefly, the cDNA encoding TEG4 scFv was amplified by PCR using the following primers: ForVHTEG4 5′-GCTACTTAAGGGTGTCCAGTGTCAGGTGCAGCTGGTGGAGTCTGG-3′ and BacVHTEG4 CCAACCTAGGACGGTCAGCTTGGTCCCTCC in order to delete 6-His and c-myc tags. The PCR fragment was then inserted into a specific transfer vector in frame with a sequence encoding a IgG1 signal peptide at the 5′ end and with a cDNA encoding a human IgG1 Fc domain with 2 extra cysteine residues at the C-terminal end.

Sf9 cells were cotransfected by lipofection with the transfer vector and purified viral DNA in the presence of 40 µl of DOTAP liposomal transfection reagent (Roche) [2]. Recombinant viruses were isolated by plaque assay and productive clones were screened by ELISA. The genomic organization of recombinant viruses was controlled by Southern blotting. Sequence of integrated genes was verified after amplification by PCR and sequencing (Eurofins Genomics, Germany).

### 2.2 Production and purification of recombinant scFv-Fc TEG4-2C

Sf9 cells were seeded at a density of 6×10^5^ cells/ml in 400 ml of serum free medium (SF900II, Life Technologies) in roller bottles and infected at a multiplicity of infection of 2 PFU (plaque forming unit) per cell. After 4 days incubation at 28 °C, supernatant was collected and secreted recombinant antibodies were purified on protein A Sepharose (GE, HealthCare). The concentration of purified antibody was determined using bicinchoninic acid (BCA) assay, as recommended by the manufacturer (Pierce) using bovine IgG (Pierce) as a standard.

### 2.3 Maleimide quantification

The number of maleimide functions expressed at droplet surface was determined using the quantitative reaction of maleimide with 2-mercaptoethanol, and the subsequent determination of the unreacted thiol with the photometric Ellman's test described by Moser [3].
