# Supplementary material for: Data on atherosclerosis specific antibody conjugation to nanoemulsions
Source: Data Brief. 2017 Oct 26;15:824–7. doi: 10.1016/j.dib.2017.10.058 (PMC5676083; doi:10.1016/j.dib.2017.10.058)
Supplement: Supplementary file 1 — Supplementary material [file mmc1.pdf]

Conflicts of Interest Statement

Manuscript title: Data on atherosclerosis specific antibody conjugaison to nanoemulsions

The authors whose names are listed immediately below certify that they have NO affiliations with or involvement in any organization or entity with any financial interest (such as honoraria; educational grants; participation in speakers' bureaus; membership, employment, consultancies, stock ownership, or other equity interest; and expert testimony or patent-licensing arrangements), or non-financial interest (such as personal or professional relationships, affiliations, knowledge or beliefs) in the subject matter or materials discussed in this manuscript.

Author names:

Geoffrey Prévot,

Martine Duonor-Cérutti,

Mélusine Larivière,

Jeanny Laroche-Traineau

Marie Josée Jacobin-Valat,

Philippe Barthélémy

Gisèle Clofent-Sanchez

Sylvie Crauste-Manciet

The authors whose names are listed immediately below report the following details of affiliation or involvement in an organization or entity with a financial or non-financial interest in the subject matter or materials discussed in this manuscript. Please specify the nature of the conflict on a separate sheet of paper if the space below is inadequate.

Author names:

This statement is signed by all the authors to indicate agreement that the above information is true and correct (*a photocopy of this form may be used if there are more than 10 authors*):

Author's name (typed)	Author's signature	Date
PREVOT		13th October 2017
Duonor-Cerutti Martine		10th October 2017
LARIVIERE		10th October 2017
LAROCHE-TRAINEAU		10th October 2017
JACOBIN-VALAT		10th October 2017
BARTHELEMY		11th oct 2017
CLOFENT-SANCHEZ		10th October 2017
CRAUSTE-MANCIET		October 10th 2017
